# Influence of MXene Composition on Triboelectricity of MXene-Alginate Nanocomposites

**DOI:** 10.1021/acsami.4c03298

**Published:** 2024-04-29

**Authors:** Bernd Wicklein, Geetha Valurouthu, HongYeon Yoon, Hyunjoon Yoo, Sathiyanathan Ponnan, Manmatha Mahato, Jiseok Kim, Syed Sheraz Ali, Jeong Young Park, Yury Gogotsi, Il-Kwon Oh

**Affiliations:** † Consejo Superior de Investigaciones Científicas (CSIC), Materials Science Institute of Madrid (ICMM), 28049 Madrid, Spain; ‡ National Creative Research Initiative for Functionally Antagonistic Nano-Engineering, Department of Mechanical Engineering, Korea Advanced Institute of Science and Technology (KAIST), Daejeon 34141, Republic of Korea; § Department of Materials Science & Engineering, and A.J. Drexel Nanomaterials Institute, Drexel University, Philadelphia, Pennsylvania 19104, United States; ∥ Department of Chemistry, Korea Advanced Institute of Science and Technology (KAIST), Daejeon 34141, Republic of Korea

**Keywords:** Ti_3_CNT_*x*_, MXene, hydrogen bonding, alginate, nanocomposite, triboelectricity, TENG

## Abstract

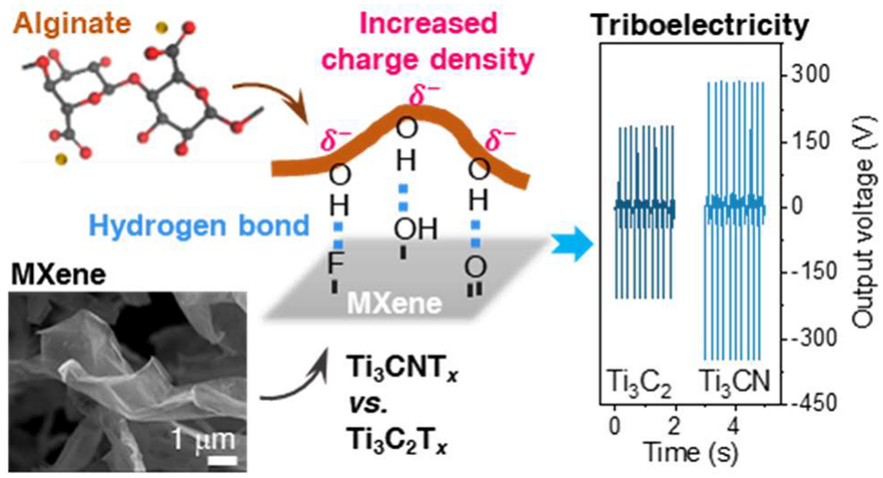

MXenes are highly versatile and conductive 2D materials that can significantly enhance the triboelectric properties of polymer nanocomposites. Despite the growing interest in the tunable chemistry of MXenes for energy applications, the effect of their chemical composition on triboelectric power generation has yet to be thoroughly studied. Here, we investigate the impact of the chemical composition of MXenes, specifically the Ti_3_CNT_*x*_ carbonitride vs the most studied carbide, Ti_3_C_2_T_*x*_, on their interactions with sodium alginate biopolymer and, ultimately, the performance of a triboelectric nanogenerator (TENG) device. Our results show that adding 2 wt % of Ti_3_CNT_*x*_ to alginate produces a synergistic effect that generates a higher triboelectric output than the Ti_3_C_2_T_*x*_ system. Spectroscopic analyses suggest that a higher oxygen and fluorine content on the surface of Ti_3_CNT_*x*_ enhances hydrogen bonding with the alginate matrix, thereby increasing the surface charge density of the alginate oxygen atoms. This was further supported by Kelvin probe force microscopy, which revealed a more negative surface potential on Ti_3_CNT_*x*_-alginate, facilitating high charge transfer between the TENG electrodes. The optimized Ti_3_CNT_*x*_-alginate nanogenerator delivered an output of 670 V, 15 μA, and 0.28 W/m^2^. Additionally, we demonstrate that plasma oxidation of the MXene surface further enhances triboelectric performance. Due to the diverse surface terminations of MXene, we show that Ti_3_CNT_*x*_-alginate can function as either tribopositive or tribonegative material, depending on the counter-contacting material. Our findings provide a deeper understanding of how MXene composition affects their interaction with biopolymers and resulting tunable triboelectrification behavior. This opens up new avenues for developing flexible and efficient MXene-based TENG devices.

## Introduction

MXenes, a family of two-dimensional (2D) transition metal carbides, oxycarbides, and/or nitrides, are represented by the general formula M_*n*+1_X_*n*_T_*x*_ (M: Ti, Mo, V, Nb, etc.; X: C, O, N; T_*x*_: −O, −OH, −F, −Cl).^1^ These materials possess exceptional properties such as metal-like conductivity and abundant active sites, making them attractive for applications in batteries and capacitors^2−5^ as well as self-powered wearables and energy harvesting devices like triboelectric nanogenerators (TENG).^6−8^ MXenes are particularly promising for TENG applications because they offer adjustable electronic, dielectric, and mechanical properties that can enhance the power output and cycle stability. For instance, a thin film of Ti_3_C_2_T_*x*_ coated on ITO can produce an open-circuit voltage (*V*_*oc*_) of 650 V and a maximum peak power of 0.5 mW.^9^ It is worth noting that research on MXenes for TENG applications has predominantly focused on Ti_3_C_2_T_*x*_.

Nevertheless, the composition of MXenes significantly influences both bulk and microscopic electrical properties, potentially impacting TENG performance. For instance, changing the element in the M site can modulate the density of states and, therefore, the electronic conduction behavior (*i.e*., metallic to semiconducting).^10^ Varying the element in the octahedral X site can control the band gap and electronegativity.^1,10^ The composition of the surface terminations (T_*x*_) influences the Fermi level and local surface dipole moments, consequently altering the work function (Φ) of MXene.^11,12^ It was shown that increasing the concentration of OH functional groups reduced Φ, while a higher concentration of O functionalities increased Φ.^13^ These deliberate modulations to the work function of TENG materials have shown the potential to boost the triboelectric voltage output,^14,15^ as recently demonstrated for Ti_3_C_2_T_*x*_ in an MXene-based TENG.^12^ Among the different techniques to modulate the surface composition of MXenes, plasma-mediated oxidation stands out as one of the most straightforward and controllable methods.^13^ This approach involves generating reactive oxygen species in the plasma, which react with the material to produce more O functionalities on the surface.

Besides, the demand for more sustainable and flexible materials for energy production has prompted the exploration of biopolymers as functional components in TENGs. In recent years, polysaccharides like cellulose, chitosan, or alginate have been explored for their suitability in triboelectric energy harvesting.^16−19^ Although occasional high output performance has been reported (300 W/m^2^) for some hydrophobic cellulose derivatives,^20^ the majority of the native, more ecofriendly biopolymers still need to catch up to conventional petroleum-based polymers in performance. This limitation stems from their low intrinsic propensity for triboelectric charge generation and retention.^21^ Nevertheless, alginate is an attractive candidate among these biopolymers. Alginate is extracted from marine biomass (brown algae) and has a linear homopolymeric structure consisting of 1–4 linked α-l-guluronic acid and β-d-mannuronic acid units.^22^ These display a high abundance of −OH groups, which are generally considered electron donors and make alginate a promising tribo-positive material.^17^ To further increase the TENG performance, appropriate fillers can be added to the biopolymers to create nanocomposites with tailored electrical properties, ensuring high short-circuit currents (*I*_*sc*_) and triboelectric potential differences.^16,23,24^ Alginate, as a water-soluble polymer, shows excellent miscibility with aqueous filler suspensions to form homogeneous composite coatings and films.^25^

MXenes emerge as ideal fillers due to their hydrophilic nature, colloidal stability, and tunable chemistry, making them a better alternative to other common fillers such as nanocarbons, silica, BaTiO_3_, TiO_2_, Ag nanowires, *etc*. Their hydrophilic nature without postsynthesis surface modification unlike in the case of other fillers guarantees good miscibility with water-based biopolymer solutions, resulting in homogeneous nanocomposite formation without phase segregation issues. In addition, they provide versatile surface chemistry for improved interaction with biopolymers, enhancing the nanocomposite’s bulk and surface conductivity.^26^ This leads to efficient charge trapping for tribocharge retention. Examples of MXene-biopolymer nanocomposites include Ti_3_C_2_T_*x*_-poly(lactic acid) and Ti_3_C_2_T_*x*_-nanocellulose films used as tribonegative components in TENG devices.^27,28^ Despite the rapid advancement of MXenes in the TENG field, a comprehensive study on how the MXene composition influences the triboelectric output of biopolymer nanocomposites is still lacking.

To address this gap, we investigated two types of MXenes, Ti_3_C_2_T_*x*_ and Ti_3_CNT_*x*_. These are among the most widespread MXenes, making their comparison important to understand how the composition of MXenes influences the triboelectric power generation of nanocomposites. We found that the higher concentration of surface oxygen and fluorine groups on Ti_3_CN significantly affects hydrogen bond interactions with the polymer matrix, thereby enhancing TENG performance. We also demonstrated the tunable TENG performance of Ti_3_CN-alginate composites through controlled oxidation. Finally, the Ti_3_CN-alginate films were tested against different dielectric polymers, including polyethylene terephthalate (PET) and nylon, to investigate their triboelectrification behavior.

## Results and Discussion

### MXene-Alginate Films as Triboelectric Nanogenerators

**Figure 1:**
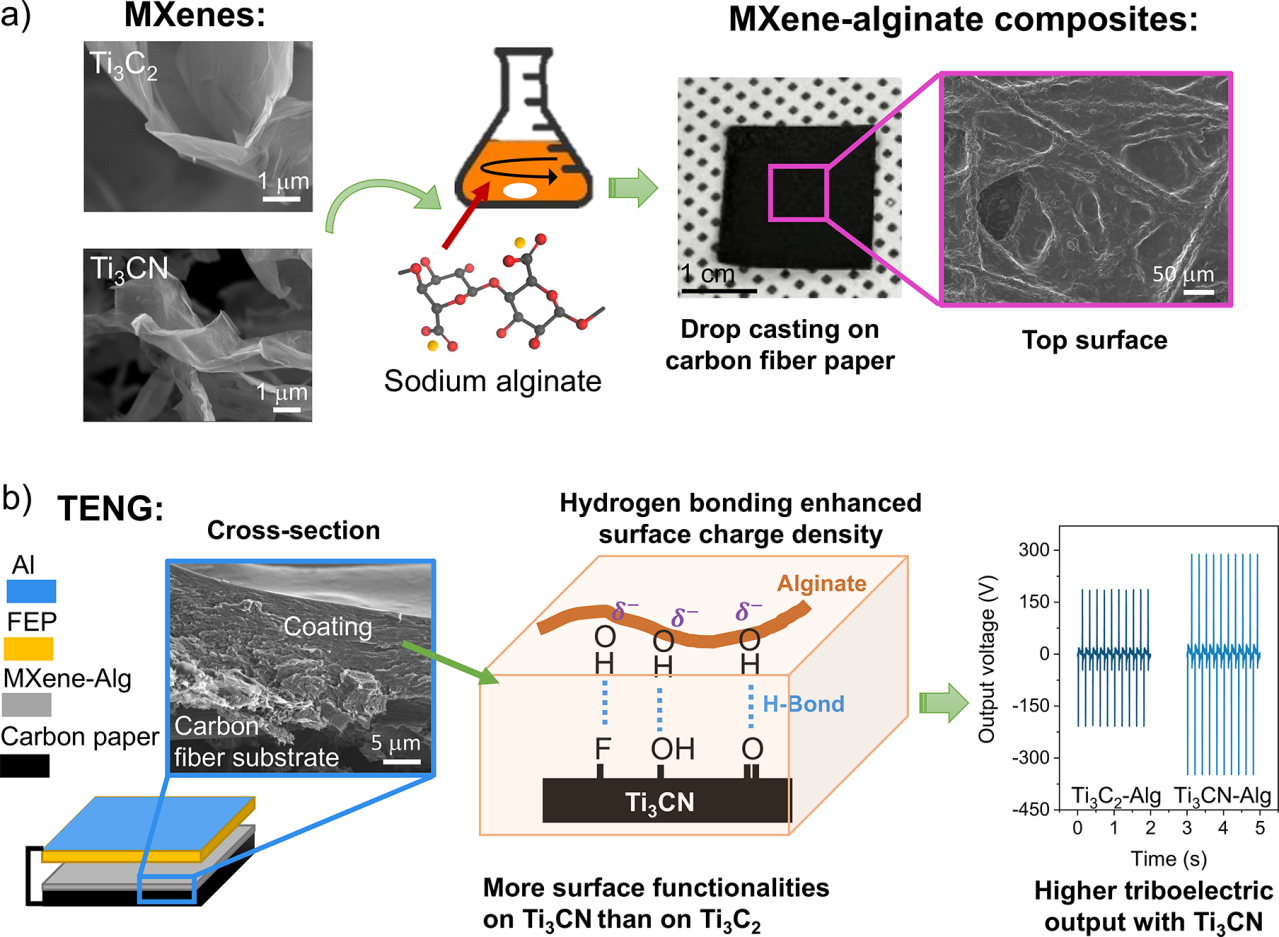
Schematic overview of the study. (a) Preparation of MXene-alginate nanocomposite; delaminated Ti_3_C_2_/Ti_3_CN flakes mixed with sodium alginate solution, followed by drop-casting onto carbon fiber paper; includes SEM images of Ti_3_C_2_ and Ti_3_CN flakes and the top surface of the resultant nanocomposite coating. (b) Illustration of a TENG device setup with an inset showing a cross-section SEM image of MXene-alginate coated carbon fiber paper, a proposed mechanism illustrating the interactions between MXene and alginate, and the resulting triboelectric voltage curves.

We tested the TENG performance of Ti_3_C_2_T_*x*_ and Ti_3_CNT_*x*_ (referred to as Ti_3_C_2_ and Ti_3_CN throughout the paper for simplicity) MXene-alginate nanocomposite coatings drop-cast on carbon fiber paper as illustrated in Figure 1a. These nanocomposites were first tested against fluorinated ethylene propylene (FEP) as the tribonegative counterpart (Figure 1b). We analyzed the structural and chemical properties of the MXene-alginate composites using microscopy and spectroscopy techniques. The performance of these nanocomposites was evaluated using Kelvin probe force microscopy and contact electrification.

**Figure 2:**
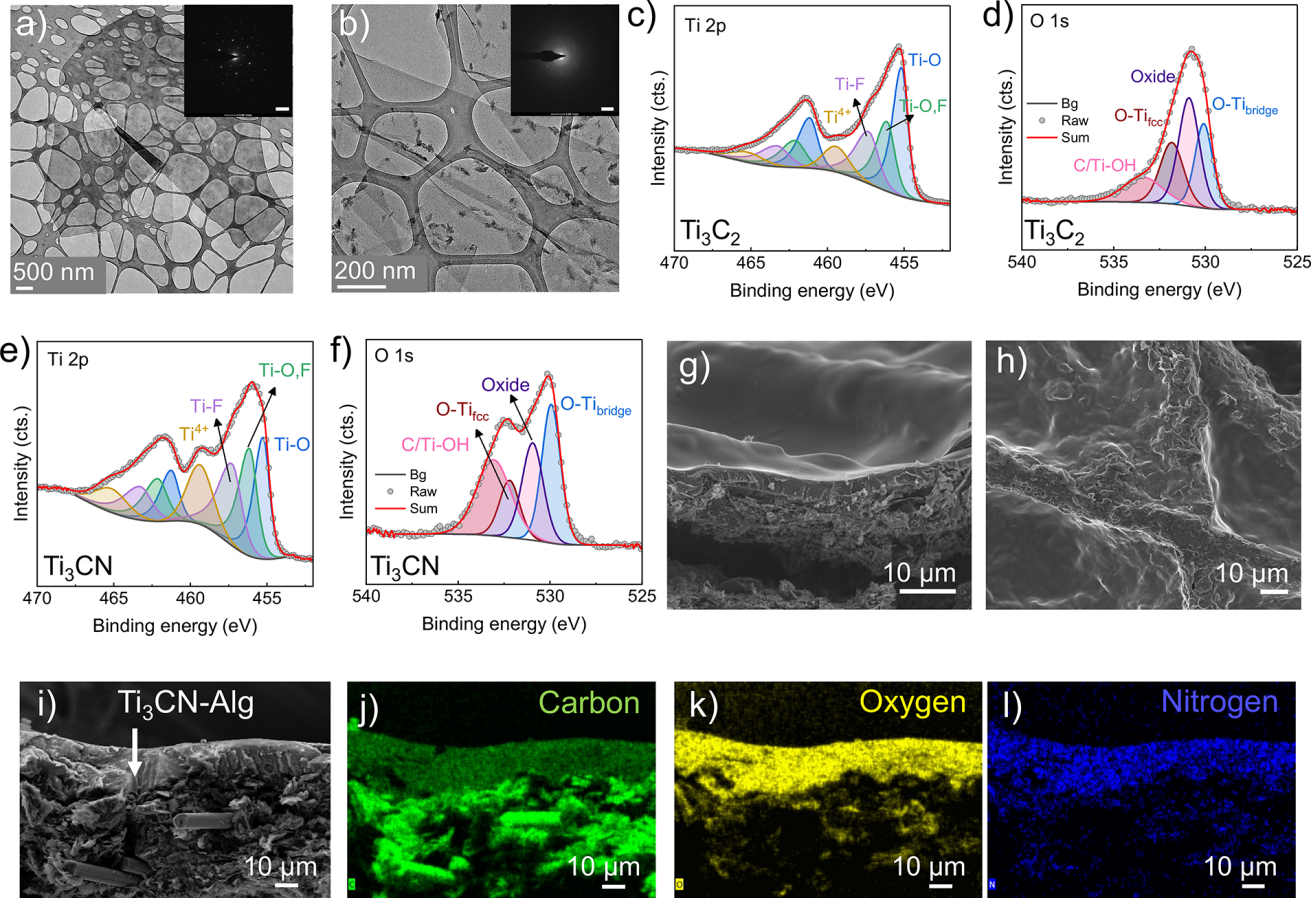
Characterization of MXenes and MXene-alginate nanocomposites. TEM images of (a) Ti_3_C_2_ and (b) Ti_3_CN, with insets showing their respective SAED patterns (scale bars refer to 5 1/nm). XPS core level spectra, including curve fittings for Ti 2p and O 1s of (c,d) Ti_3_C_2_ and (e,f) Ti_3_CN, respectively. SEM images of (g) the cross-section and (h) the surface of the Ti_3_CN-Alg coating on carbon fiber paper. EDS elemental maps obtained from (i) Ti_3_CN-Alg coating displaying (j) carbon, (k) oxygen, and (l) nitrogen distribution.

This study utilized fully delaminated Ti_3_C_2_ and Ti_3_CN MXenes as fillers to prepare MXene-alginate nanocomposites. Transmission electron microscopy (TEM) (Figure 2a,b) and atomic force microscopy (AFM) (Figure S1) showed a flake-like morphology of these MXenes with a lateral dimension of about 5 μm and a thickness comparable to one and two layers of MXene flakes (1.8–3.8 nm).^29^ The hexagonal symmetry structure of the MXenes was further confirmed by selected area electron diffraction (SAED) micrographs (Figure 2a,b, insets). It is apparent that Ti_3_C_2_ (sharp spots) has a higher degree of crystallinity than Ti_3_CN (diffused diffraction rings), as inferred from their respective diffraction patterns. This is often observed for carbonitride MXenes and results from the MAX phase precursor and harsh synthesis conditions.^30^ X-ray diffraction (XRD) additionally confirms the successful preparation of MXenes from their respective MAX phases, as they do not show the (014) peak of the MAX phase and only exhibit (00*l*) reflections, typical of delaminated MXenes (Figure S2).^29,31^

**Figure 3:**
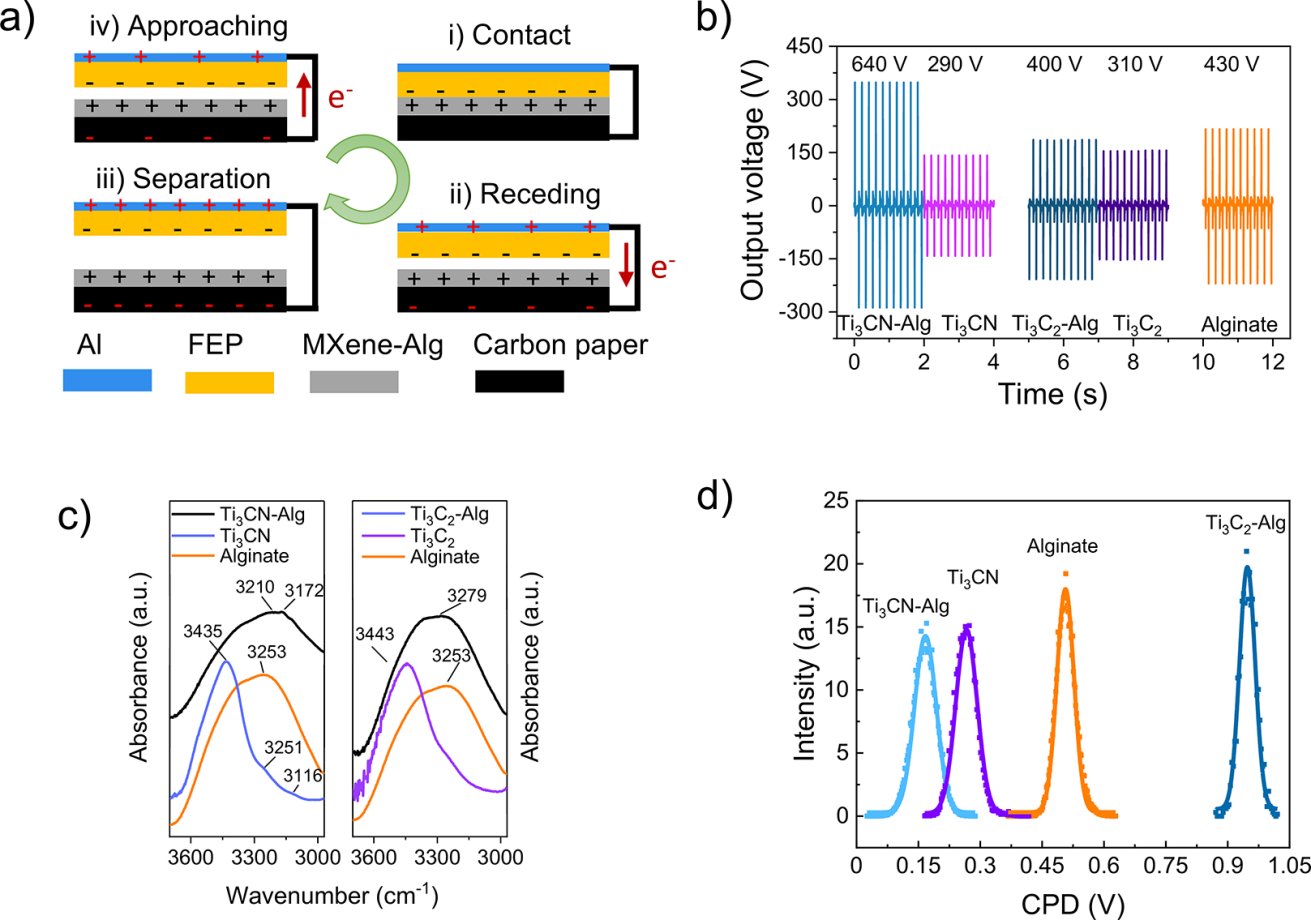
Influence of the MXene composition on the hydrogen bond interactions and triboelectric behavior. (a) Illustration of the TENG working principle, (b) triboelectric output voltage curves of alginate, the MXenes, and the MXene-alginate composites, (c) FTIR spectra of the OH band region, (d) contact potential difference (CPD) distribution curves.

Raman and Fourier transform infrared (FTIR) spectra (Figure S3) show the presence of surface functional groups, typically F, O, and OH.^32^ The lower intensity of the corresponding IR absorbance bands in Ti_3_C_2_ (Figure S3b) suggests that this MXene has fewer polar bonds and/or fewer terminal groups than Ti_3_CN. Analyzing XPS core level spectra (Figures 2c–f and S4) provided estimates of the elemental composition of Ti_3_CN and Ti_3_C_2_ MXenes: Ti_3_C_1.47_N_0.98_F_1.52_O_1.49_ and Ti_3_C_1.98_F_1.12_O_1.08_, respectively (Tables S1 and S2). Hence, Ti_3_CN has higher fluorine and oxygen contents, which agrees with the IR data. It is clear from O 1s spectra that surface C/Ti–OH groups are more abundant in Ti_3_CN (6.1 at%) than in Ti_3_C_2_ (3.7 at%) (Figure 2d,f). Additionally, the oxide (Ti^4+^) to the overall Ti content is higher for Ti_3_CN (20%) than Ti_3_C_2_ (12%). The aqueous MXene suspensions exhibited zeta potentials of −52 mV (Ti_3_C_2_) and −30 mV (Ti_3_CN). The less negative zeta potential of Ti_3_CN in suspension is possibly due to the surface passivation by oxide and amine NH_*x*_ species (see XPS core level Ti 2p, O 1s, and N 1s spectra in Figure 2c–f and Supporting Information Figure S4f).^33^ Nevertheless, both zeta potentials provide high colloidal stability necessary for forming homogeneous mixtures with the sodium alginate (Alg) solution. These MXene-alginate suspensions were drop-cast on carbon fiber paper, forming 5–10 μm thick nanocomposite coatings on the external surface as seen in the cross-section scanning electron microscopy (SEM) image (Figure 2g) and top surface SEM image (Figure 1a). Conducting carbon paper was chosen as a support because it serves as a current collector and back-electrode in the TENG (Figure 3a). The wettability of the carbon paper surface was increased by O_2_ plasma activation before drop-casting, which ensured the formation of homogeneous coatings. In contrast, the back side of the paper remained unmodified and conducting (5 Ω resistance). Figure 2g,h shows representative SEM images for Ti_3_CN-alginate coatings, while images of the Ti_3_C_2_-alginate coatings are provided in Figure S5 for comparison. A closer inspection of the surface indicates that the coating adopted the granular and rough texture of the underlying fiber mat (Figure 2h). Comparison with the uncoated carbon fiber paper (Figure S6) indicates that the MXene-alginate coating only slightly reduced the roughness of the surface, given its small thickness. Finally, elemental mapping of the cross-section with energy-dispersive X-ray spectroscopy (EDS) allows a clear distinction between the coating and the substrate using the carbon, oxygen, and nitrogen signals (Figure 2j–l). The carbon paper substrate has a higher carbon content than the composite, which also contains oxygen, among other elements. The sodium alginate polymer is rich in oxygen (molecular structure NaC_6_H_7_O_6_), and therefore, the composite coating shows a distinctive oxygen signal. The MXene (Ti_3_CN in this case) contributes to the oxygen and nitrogen signals, as revealed by the XPS compositional analysis above. When MXene was deposited on the carbon paper without the polymer matrix, delamination of the film could be observed (Figure S7), which suggests that the alginate has a significant adhesive effect and improves the mechanical integrity of the film. The electrical conductivity of the MXene-alginate nanocomposites was determined on solvent-cast, freestanding films (Figure S8a) because the porosity of the carbon fiber paper makes an accurate four-probe measurement on the coatings difficult. Nanocomposite films containing Ti_3_C_2_ showed increasing conductivity with filler content (0.9 mS/cm at 2% and 88 mS/cm at 5%), while the Ti_3_CN nanocomposites remained nonconducting. The conductivity of the MXenes was determined on vacuum-filtered films (Figure S8b), where Ti_3_C_2_ displayed 12 300 S/cm and Ti_3_CN 2 400 S/cm. These values agree well with the typical conductivities of these MXenes, where carbonitrides tend to have lower values, possibly related to their reduced crystallinity.^29^ Hence, this may explain the lower electrical conductivity of the Ti_3_CN-Alg composite films.

The TENG device in this study was operated in contact-separation mode. Herein, the side of the carbon paper displaying the MXene-Alg film is contacted with a dielectric polymer film (FEP) glued on aluminum tape. In this setup, the carbon paper and Al tape act as back-electrodes connected to the outer circuit (Figure 3a). Through contact electrification, charges of opposite signs are generated on each surface.^34^ When the films are separated, charges are induced in the back-electrodes, which drive a current through the outer circuit. At complete separation, the system enters again the electrical equilibrium, where charges on the back-electrodes balance the surface charges. When the films are approaching, the electron flow is reversed until the surfaces are again in contact. Figure 3b shows the triboelectric voltage curves of Ti_3_CN-Alg, Ti_3_CN, and alginate. Plain Ti_3_CN and alginate were deposited on carbon paper under the same conditions as Ti_3_CN-Alg. The results show that the composite produces the highest output voltage (640 V), whereas Ti_3_CN alone produces 290 V and alginate about 430 V. This finding suggests an apparent synergistic effect, where the composite generates a higher voltage than each of the plain components.

### Hydrogen Bond Interactions and Surface Potential Measurements

One possible reason for the observed synergistic effect is specific interactions between the alginate matrix and the Ti_3_CN filler that enhance the electron donor behavior of alginate. Polysaccharides like alginate are known to donate electrons in the contact electrification process with strong electron-withdrawing (*i.e.*, tribonegative) materials such as fluorinated ethylene propylene.^21^ Besides the carbon atoms, the oxygen atoms on the surface are suggested to provide tribo-charge.^35^ We investigated how the interaction with the surface fluorine and oxygen atoms in Ti_3_CN can alter the electron density of oxygen in the OH groups of alginate, leading to an increase in the tribo-charge donation. FTIR spectra show the stretching vibration range of OH groups in alginate, Ti_3_CN, and 5%Ti_3_CN-alginate (Figure 3c). A noticeable redshift of the vibration frequency is observed from 3253 cm^–1^ in alginate to 3172–3210 cm^–1^ in the composite, while the OH vibrations in Ti_3_CN are centered at 3435 cm^–1^. This shift is typically attributed to hydrogen bond (HB) interactions of OH with HB acceptors having a lone electron pair.^36,37^ In particular, F atoms are strong HB acceptors that can attract the hydrogen atom, elongate the O–H bond length, which results in lower vibration frequency (*i.e*., redshift), and enhance the electron density of the oxygen atom by transferring the high electron density of F (MXene) to O (alginate).^38^ In addition, the high oxygen content (20 at%), which includes −O, −OH, H_2_O, TiO_2_, combined with the smaller amount (0.9 at%, Table S1) of amino groups of Ti_3_CN, are expected to play a significant role in the strong HB interactions with alginate.

It should be noted that any possible shift of the fluorine bands (*i.e*., Ti–F, C–F) due to HB interactions is camouflaged by the overlapping alginate bands (Figure S9). As a consequence of these interactions, the increased charge density contributes to more charge transfer to FEP during contact, thereby enhancing the open-circuit voltage, *V*_*OC*_, according to well-established models (*V*_*OC*_ = σ × *x*(*t*)/ε_*o*_), where σ is the surface charge density, *x*(*t*) is the interlayer distance, and ε_o_ is the vacuum permittivity.^39^ The role of HB in increasing the TENG output was also described in other composites, such as nylon-graphene oxide, where HB was shown to influence the surface potential of the composite.^40^

Kelvin probe force microscopy (KPFM)^18,19^ was used to examine the surface potential (contact potential difference (*V*_*CPD*_) profiles) of alginate, Ti_3_CN, and Ti_3_CN-Alg (Figure 3d). The measurements suggest that adding Ti_3_CN to alginate reduces the surface potential from 500 to 150 mV. Surface potential images of the samples are provided in Figure S10. A reduced surface potential indicates a negatively charged surface and would support the conclusions drawn from the FTIR data. Another reason for the lower surface potential of the composite can be charges trapped on the Ti_3_CN filler. MXenes have been reported as efficient charge trappers due to their high electronegativity and abundant surface terminations.^26^ Surface charge traps are known to increase the work function (ϕ) by lowering the Fermi level.^41^ The determined *V*_*CPD*_ is related to the work function of the sample through ϕ_*s*_ = ϕ_*p*_ – *eV*_*CPD*_, where ϕ_*p*_ is the work function of the probe and *e* is the elemental charge. Hence, the reduced *V_CPD_* of Ti_3_CN-Alg indicates an increase in the sample work function in agreement with the charge trap notion of conducting fillers,^23^ in addition to the effect of HB interactions. Trapped charges have also been reported to enhance the triboelectric voltage by increasing surface charge density.^23,41^ In addition, the incorporation of the flake-like MXenes makes the surface of Ti_3_CN-Alg slightly rougher (RMS = 176 nm) than that of alginate (RMS = 144 nm) as observed by SEM (Figure S11a) and determined with a 2D profiler (Figure S11b). Surface roughness and nano/microstructures commonly increase the triboelectric voltage output.^42^

On the other hand, adding Ti_3_C_2_ (2 wt %) to the alginate matrix resulted in a voltage output of 400 V, slightly below the plain alginate value (Figure 3b). Pure Ti_3_C_2_ and alginate delivered 310 and 430 V, respectively. This indicates that there was no synergistic effect between this MXene and alginate, starkly contrasting the Ti_3_CN system. The absence of a redshift in the IR spectra suggests that there was no pronounced HB interaction between Ti_3_C_2_ and the alginate matrix (Figure 3c). One possible reason for this could be the significantly lower content of O and F groups in Ti_3_C_2_ compared to Ti_3_CN. As a result, fewer HB acceptors were available for interaction with alginate, which would create less electron charge density on the composite surface. This rationale was validated by the recorded CPD curves indicating a higher surface potential of Ti_3_C_2_-Alg (Figure 3d). In summary, strong HB interactions between Ti_3_CN and alginate may originate from the electronegative nitrogen conductive core as well as fluorine, oxide, and amine NH_*x*_ species found in Ti_3_CN. This resulted in a more negative surface potential on the composite than Ti_3_C_2_-alginate.

### Controlled Oxidation of Ti_3_CN-Alginate

**Figure 4:**
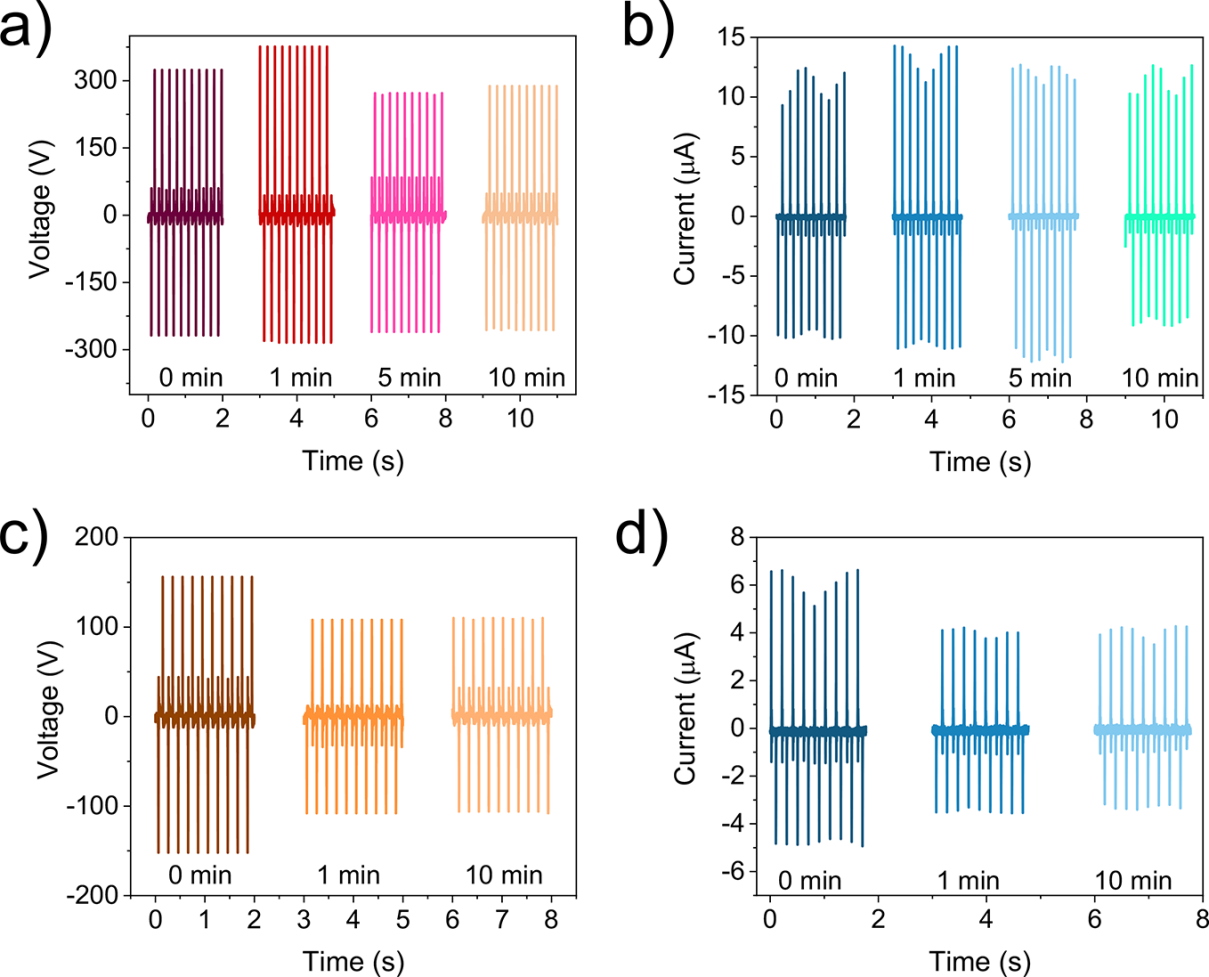
Influence of plasma oxidation on triboelectric performance. Triboelectric voltage and current output of Ti_3_CN-alginate (a,b) and alginate (c,d) films subjected to varying durations of oxygen plasma treatment.

The role of oxygen species concerning the TENG performance was further investigated. To this end, Ti_3_CN-alginate films were exposed to oxygen plasma for 1–10 min. The triboelectric voltage and current curves of these films (Figure 4a,b) demonstrate a pronounced increase after 1 min plasma oxidation with maximum values of 670 V and 15 μA. However, upon prolonged treatment, the output decreased again (Figure S12). Alginate films were also subjected to the same process for comparison, and the results showed that oxygen treatment did not increase the TENG performance (Figure 4c,d). This indicates that MXene is crucial for the output enhancement effect upon plasma oxidation. It has been observed that the controlled oxidation of Ti_3_C_2_ by O_2_ plasma generates abundant Ti–O groups and TiO_2_ clusters by breaking Ti–C bonds.^13,26,43^ In fact, it was also found that the OH abundance increased after 1 min.^13^ In contrast, at more prolonged plasma exposure, the number of Ti–O and TiO_2_ groups became more dominant. Both experimental and theoretical (DFT) studies indicate that the composition of the surface terminations influences the work function of MXenes.^12,13,43−45^ It has been found that the work function decreases with more −OH terminations and increases with higher =O and −F content. A reduced work function generally makes materials more tribopositive, enhancing the propensity to donate electrons, which increases the *V*_*oc*_ of a TENG.^15^ This would explain the observed trend with increased voltage output at short oxidation time, while with longer oxidation, the =O content increases, and, as a consequence, the work function increases as well. It was also shown in DFT calculations that the terminations have a stronger influence on the work function than the M and X species owing to the formed surface dipoles.^44,46^ This implies that plasma oxidation on Ti_3_CN should have similar effects as on Ti_3_C_2_ reported in the literature.^13,26,43^ In addition, the modified surface oxygen composition of Ti_3_CN should affect the HB interactions with the alginate matrix. An increased oxygen content would increase HB interactions, resulting in more polarized oxygen atoms of alginate and, consequently, a larger surface charge and increased triboelectric voltage. It has been argued that the hydroxyl groups are formed initially from residual water molecules in the MXene structure reacting with freshly cleaved Ti–C groups. These findings align with other reports suggesting that (controlled) oxidation of MXenes is beneficial for improving certain functional properties including triboelectrification and electron mobility.^12,26,43^ It is important to note that the higher electrical conductivity of Ti_3_C_2_-Alg films did not contribute to enhanced triboelectric performance. This effect was possibly outweighed by the composition effect, as elaborated above.

### Triboelectrification Behavior

**Figure 5:**
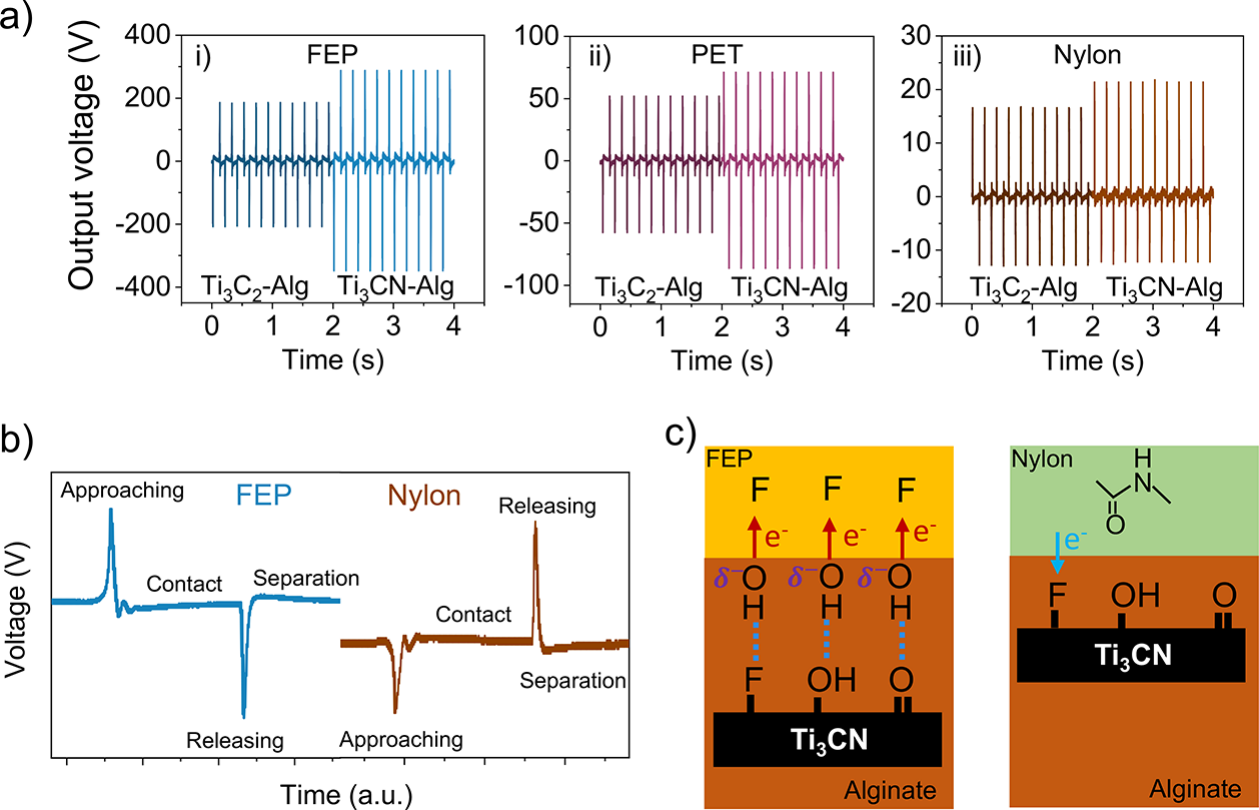
Triboelectric behavior with different counter dielectric layers. (a) Voltage curves of the nanocomposites contacted with FEP (i), PET (ii), and nylon (iii). (b) Enlarged voltage curves of Ti_3_CN-alginate with FEP and nylon. (c) Illustration of contact electrification of Ti_3_CN-Alg with FEP and nylon, respectively, indicating the triboactive functional groups in each polymer.

Next, the MXene-alginate films were tested against three dielectric polymers—fluorinated ethylene propylene (FEP), polyethylene terephthalate (PET), and nylon—each exhibiting different triboelectrification behavior according to their position in the triboelectric series.^21^ FEP is a highly tribonegative material with a strong tendency to withdraw electrons, PET is a weaker tribonegative material, and nylon is a tribopositive material with a strong tendency to donate electrons. The output voltage curves of the nanocomposite films contacted with FEP, PET, and nylon demonstrate that Ti_3_CN-Alg consistently outperformed Ti_3_C_2_-Alg in all cases, and the output varied with the chosen polymer (Figure 5a). Contacting with FEP produced a significantly higher voltage (Ti_3_C_2_-Alg: 430 V; Ti_3_CN-Alg: 640 V) than with PET (105 V; 155 V) and nylon (15 V; 34 V). The higher triboelectric voltage is due to the strong electron withdrawal caused by the fluorine atoms in the FEP structure. The direction of electron flow is reversed for nylon compared to FEP when tested against Ti_3_CN-Alg, as evident from their voltage curves (Figure 5b). For instance, positive voltage signals were recorded for FEP approaching Ti_3_CN-Alg, whereas a negative signal was produced during the nylon approach. This implies that charges are transferred from nylon to the nanocomposite and vice versa in the case of FEP (and PET) (Figure 5c). The fluorine atoms on Ti_3_CN flakes located at or slightly below the contact surface are likely responsible for withdrawing electrons from nylon, similar to the behavior of fluorine in FEP. Consequently, it can be inferred that these MXenes can function both as tribonegative and tribopositive materials, deviating from their conventional use as tribonegative electrodes or fillers in TENGs.^8,9^

### Performance Characterization of Ti_3_CN-Alginate TENG

**Figure 6:**
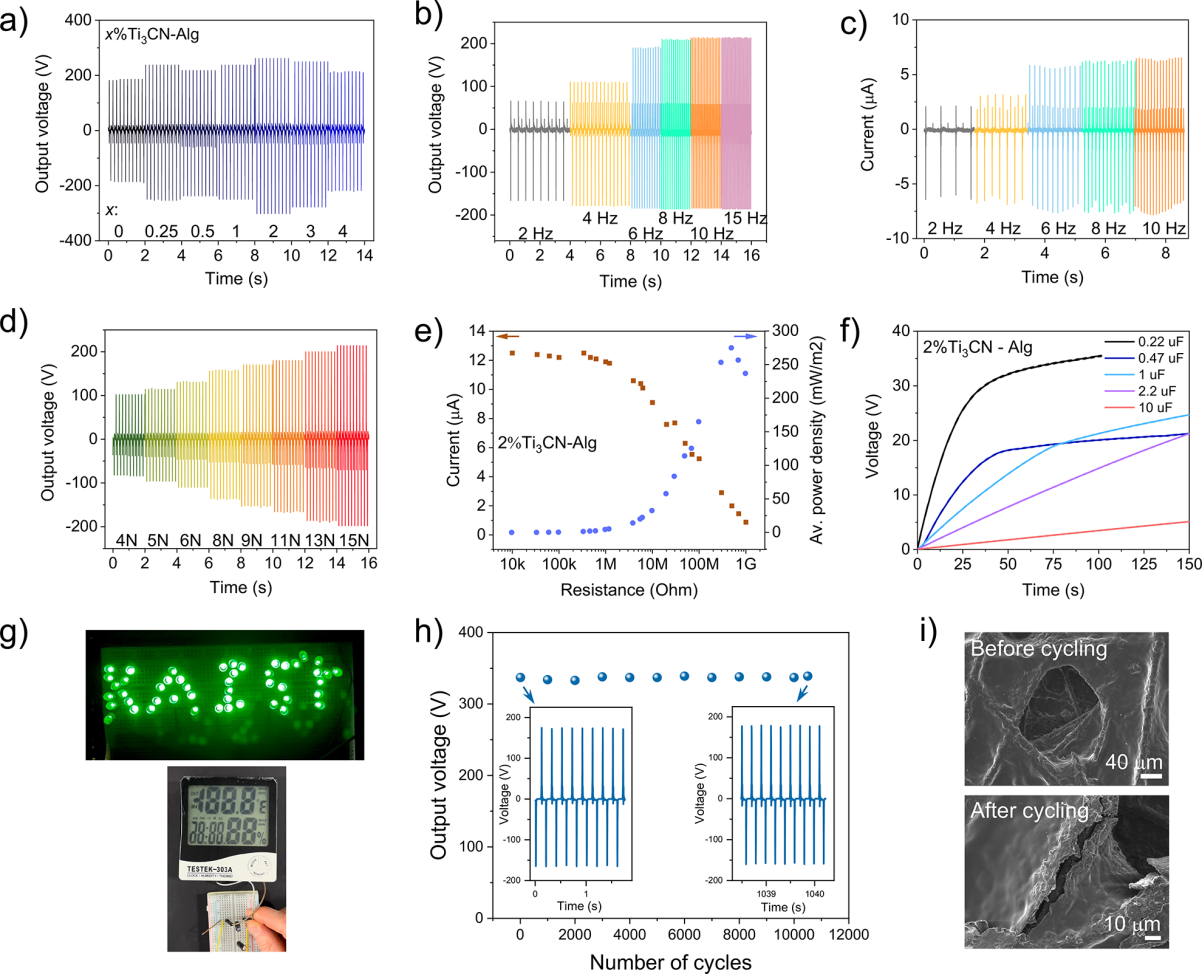
Triboelectric power generation with respect to excitation conditions. (a) Voltage output of *x*% Ti_3_CN-alginate (x = 0–4). (b) Voltage and (c) current output as a function of the contact frequency and (d) voltage vs. contact force of 2% Ti_3_CN-alginate, respectively. (e) Power load and current curve, (f) capacitor charging curves, (g) powering of LEDs and a small device with 2%Ti_3_CN-alginate, (h) cycling stability, and (i) SEM images of 2%Ti_3_CN-alginate before and after the cycling test.

Given the higher output from Ti_3_CN-alginate, the triboelectric performance of these nanocomposites was characterized in more detail. Varying the concentration of Ti_3_CN increased the output voltage, showing a maximum at 2% Ti_3_CN, after which it decreased again (Figure 6a). Such behavior is commonly observed in nanocomposite TENGs and is attributed to competing influences. While surface roughness and dielectric constant typically increase with filler concentration and generally enhance the voltage, filler agglomerations, short-circuiting, and reduced effective contact area may dominate at higher concentrations and tend to reduce the triboelectric output.^23^ Because of this voltage maximum, the Ti_3_CN filler concentration used in the present study was generally 2% unless stated otherwise. Increasing the contact frequency and force enhanced the voltage and current output (Figure 6b–d) in agreement with the expected behavior of TENGs.^47^ The time-averaged power density, *P*_*av*_, and short-circuit current, *I*_*sc*_, were measured across resistance loads (Figure 6e). The current output is 12 μA, and the maximum *P*_*av*_ is 0.28 W/m^2^ at a load of 500 MΩ, while the instantaneous power density is 6.8 W/m^2^ at 100 MΩ. This value is superior to or on par with other MXene-biopolymer composite TENGs reported in the literature, such as Ti_3_C_2_ in alginate/ecoflex (0.05 W/m^2^, 1.0 μA, 200 V),^48^ in cellulose nanofiber (0.5 W/m^2^, 5.5 μA, 300 V),^49^ in carboxymethyl cellulose (0.4 W/m^2^, 0.8 μA, 120 V),^50^ and in poly(lactic acid) (0.5 W/m^2^, 22 μA, 100 V)^27^ composites, respectively. It should be noted that many publications do not explain how the power density was calculated, **i.e.**, the time-averaged or the instantaneous power. The latter gives higher, albeit technically irrelevant, overestimated values, unlike the *P*_*a*v_ determined in this study. The TENGs using MXene-biopolymer composites are still scarce and mainly use Ti_3_C_2_ composites, while Ti_3_CN-based biopolymer composites are largely missing in this context. This emphasizes the significance of expanding the range of usable MXenes. Furthermore, the Ti_3_C_2_ content in these composites ranges from 2 to 80 wt % in the optimized cases. In this work, with only 2 wt % of MXene, the optimum TENG performance was achieved, underscoring the importance of a homogeneous dispersion facilitated by the excellent miscibility of MXene and alginate solutions. The charging performance of Ti_3_CN-alginate was also examined with capacitors ranging from 0.22 to 10 μF (Figure 6f). While 0.22 and 0.47 μF capacitors are fully charged after 45 s, the energy stored in a 1 μF capacitor after 100 s is 0.23 mJ. The Ti_3_CN-alginate TENG could power small electric devices such as a temperature sensor or an array of 50 LEDs (Figure 6g). The long-term cycling stability of the nanocomposite was evaluated, showing no voltage decay after 10 500 contact-separation cycles (Figure 6h). This indicates that the material is not prone to deterioration, which would have caused a performance decrease. SEM investigation of the film surface before and after the cycling experiment suggests only slight cracking and delamination of the Ti_3_CN-alginate coating (Figure 6i), which was not severe enough to affect the TENG performance. These results agree with the mechanical reinforcement effect of MXenes in polymer composites improving wear and impact resistance, which is attributed to outstanding mechanical properties and lateral sliding of the MXene sheets.^51^

In prior work, researchers mainly focused on Ti_3_C_2_ for TENG applications, paying less attention to the vast chemical diversity of MXenes to improve performance further. Our results show that Ti_3_CN is a more effective triboelectric filler than Ti_3_C_2_, with output voltages of 640 and 420 V, respectively. This finding agrees with other studies of MXene TENGs, where Ti_3_CN was more effective than Ti_3_C_2_ in enhancing the surface charge density of poly(vinylidene difluoride) through strong electrostatic interactions with the polymer.^52^ Similarly, chemical modification of Ti_3_C_2_ with nitrogen groups also increased the dipolar polarization of the nanocomposite, resulting in higher energy output.^53^ While reports on niobium- and vanadium-based MXene TENGs are rarer still, we showed exemplarily how tuning the composition of MXenes during synthesis and postsynthesis can be a versatile lever to improve the triboelectric performance of biopolymers. We anticipate that our findings will promote the exploration of various MXenes beyond Ti_3_C_2_ in TENG applications and provide guidance for enhancing the triboelectric performance of MXene-based devices.

## Conclusions

We have demonstrated that the composition of MXenes, Ti_3_C_2_T_*x*_ and Ti_3_CNT_*x*_, significantly influences the triboelectric behavior of alginate nanocomposites. Typically, MXenes are used as tribonegative (electron-withdrawing) materials in TENGs due to their fluorine terminations. However, we found that MXenes can also be highly effective tribopositive materials. Particularly, Ti_3_CNT_*x*_ fillers improved the triboelectric output of alginate compared to Ti_3_C_2_T_*x*_, rendering it more tribopositive (electron donor behavior). The higher O and F content of Ti_3_CNT_*x*_ versus Ti_3_C_2_T_*x*_ resulted in stronger hydrogen bond interactions with alginate −OH groups, increasing the local charge density and effectively enhancing the triboelectric power generation. The optimized Ti_3_CNT_*x*_-alginate nanogenerator produced 670 V, 15 μA, and 0.28 W/m^2^, making it suitable for charging low-power devices. Additionally, the raw materials used in this nanogenerator are biocompatible and sustainable, in contrast to commonly used perfluorinated TENG materials. We also found that the modulation of oxygen functionalities through plasma oxidation plays a crucial role in improving TENG performance. Furthermore, our findings revealed that MXenes could function as tribopositive and tribonegative fillers due to electron-donating (O) and electron-withdrawing (F) surface groups. This versatility may lead to future research using the extensive MXene composition library to create truly antagonistic materials.

## Materials and Methods

### Materials

Ti, TiC, Al, AlN, graphite, HCl, LiCl, and alginic acid (sodium salt form, low-viscosity grade), were procured from Sigma-Aldrich, while carbon fiber paper (AvCarb P50) was acquired from Fuel Cell Store.

### MAX Phase Synthesis

Preparation of the MAX phases followed procedures described previously.^29,31^ For the preparation of the Ti_3_AlC_2_ MAX phase, precursor powders of TiC, Ti, and Al were combined in stoichiometric proportions of 2:2.2:1.25. This mixture was subjected to ball milling, utilizing yttrium-stabilized zirconium balls (3 mm in diameter) with a powder-to-ball weight ratio of 1:2 in high-density polyethylene bottles. Milling was performed at a speed of 70 rpm for 15 h. Subsequently, the milled powder mixture was transferred to alumina crucibles for sintering at 1380 °C for 2 h under a continuous argon gas flow, with a heating and cooling rate of 3 °C/min. After sintering, the resulting ceramic block was milled using a titanium carbide bit and further ground using a porcelain mortar and pestle. To obtain particles with a size of less than 38 μm, the powder was sieved through a 400-mesh sieve. An additional step involved acid-washing the MAX phase using 9 M hydrochloric acid for 24 h to eliminate intermetallic compounds and unreacted metals. The acid-treated powders were collected via vacuum filtration using a 5 μm polycarbonate filter membrane, followed by thorough rinsing with deionized (DI) water until a pH greater than 6 was achieved. The collected MAX phase powder was subsequently dried in a vacuum oven at 60 °C overnight.

To prepare the Ti_3_AlCN MAX phase, Ti, AlN, and graphite powders were combined in stoichiometric proportions of 3:1:1. The powder mixture was then subjected to ball milling as described above. The milled powder mixture was sintered at 1500 °C for 2 h under a continuous argon gas flow, employing a heating and cooling rate of 3 °C/min. The subsequent steps in the synthesis process remained consistent with those described above.

### MXene Synthesis

The Ti_3_C_2_T_*x*_ and Ti_3_CNT_*x*_ MXenes were prepared through etching and delamination of the corresponding MAX phases, Ti_3_AlC_2_ and Ti_3_AlCN, respectively. It was carried out following the methods described previously.^29,31^*Etching of MAX phase* – 1 g of MAX phase powder was slowly added to a high-density polyethylene bottle containing an etchant solution of 2 mL of HF (49% solution), 12 mL of HCl (36% solution), and 6 mL of DI water under gentle stirring. After adding MAX phase powder, the stirring speed and the temperature were set to 360 rpm and 35 °C, respectively, and allowed to stir for 24 h. After the etching reaction was complete, the resulting mixture was centrifuged at 3500 rpm for 5 min. The supernatant was discarded, and the sediment was dispersed with DI water. This washing process was repeated until the pH of the supernatant reached a value greater than 6, and then, the sediment was collected. *Delamination of MXene* – The collected sediment was used for the delamination process. It was resuspended in a 20 mL mixture composed of 1 g of LiCl in DI water, followed by stirring for 24 h at 360 rpm and 35 °C, respectively. Subsequently, the mixture was transferred to a centrifuge tube and centrifuged at 3500 rpm for 10 min. The supernatant was discarded, and the sediment was resuspended using DI water. This step was repeated until the supernatant became dark, indicating successful delamination. The supernatant was collected, while the sediment was resuspended in DI water and shaken for 5 min. The suspension was then centrifuged at 3500 rpm for 10 min, and the supernatant was once again collected. The resuspension, shaking, and centrifugation process was repeated until all the MXene was collected. Colloidal suspensions with a concentration of 8 mg/mL Ti_3_C_2_T_*x*_ and 10 mg/mL Ti_3_CNT_*x*_ (subsequently denoted as Ti_3_C_2_ and Ti_3_CN for simplicity) were obtained and stored at 4 °C until further use but not longer than 1 month.

### MXene-Alginate Coatings

MXene aliquots were dispersed in a 2% (w/v) alginate solution to render MXene concentrations of 0–5% (w/w) with respect to alginate. The mixtures were vortexed and then stirred for 1 h at room temperature before drop-casting 1 mL of the mixture on the activated side of carbon fiber paper (covering 2 × 2 cm^2^ area). This side was activated by oxygen plasma using a Covance vacuum plasma instrument from Femto Science (Republic of Korea) at 150 W for 5 min. Following drop-casting, the samples were oven-dried at 40 °C. If not stated otherwise, Ti_3_C_2_-Alg and Ti_3_CN-Alg refer to the 2% (w/w) MXene concentration for simplicity. Some Ti_3_CN-Alg films were also exposed to oxygen plasma (150 W, 10 sccm O_2_ flow) for 1–10 min to achieve controlled surface oxidation.

### Characterization

Microstructural analysis by X-ray diffraction (XRD) was performed with a Rigaku Smartlab instrument using Cu K_α1_ radiation with a step size of 0.01° and 1 s/step. Raman spectra were recorded on a Horiba LabRAM HR Evolution using the 633 nm laser line and a 50x objective. Spectra were smoothed with a Savitzky-Golay filter (seven points). Fourier transform infrared (FTIR) spectra were recorded on a Nicolet iN10MX instrument from Thermo Fisher Scientific.

Morphological information was obtained from scanning electron microscopy (SEM) using a JEOL JSM-IT800 after 3.4 nm osmium sputtering of the sample surface and transmission electron microscopy (TEM) on an FEI Talos instrument operated at 200 kV. Energy-dispersive X-ray spectroscopy (EDS) was coupled to the SEM, and selected area electron diffraction (SAED) was performed with the TEM.

Chemical analysis was carried out by X-ray photoelectron spectroscopy (XPS) measurements, which were performed using the Nexsa G2 system from Thermo Scientific with monochromatic Al K_α_ (1486.6 eV) radiation with a 400 μm spot size. The materials for the measurements were affixed onto conductive double-sided carbon tape. Pass energy and step size were set at 50 and 0.05 eV, respectively. No Ar^+^ sputtering was performed before the XPS acquisition. The spectra were analyzed using CasaXPS V2.3.19 software, and the binding energy scale of pristine MXenes and MXene alginate nanocomposites were calibrated using the C–Ti and C–C/C–H C 1s binding energy at 282 and 284.8 eV, respectively. A Shirley background was applied to all core spectra.^54,55^

Electrical properties were characterized with a Malvern Zetasizer Nano S and a four-probe instrument (Model 4040, MS Tech) connected to a source meter (2400 Keithley). For the electrical conductivity measurements, freestanding films were prepared by solvent casting MXene-alginate suspensions (dried at 50 °C) and by vacuum filtration of MXene suspensions using a Celguard 3401 membrane. The surface potential was determined by Kelvin probe force microscopy (KPFM) using an atomic force microscope (Keysight 5500). The probe was a conductive, noncontact cantilever (PPP-EFM, Nanosensors) coated with Pt/Ir and a resonance frequency of 71.4 K Hz. To determine the work function of the tip, a contact potential difference (CPD) measurement was taken on a polycrystalline gold inert reference material before and after each sample measurement. The topography and CPD of the samples were then measured through KPFM techniques. When operating in KPFM mode, the DC bias eliminates any interaction caused by the potential difference between the tip and the sample, which is then measured as CPD. The *V*_*CPD*_ value is calculated as , where Φ_*tip*_ and Φ_*sample*_ denote the respective work function values of the tip and sample, while *e* represents the charge of an electron. The surface roughness of freestanding films was measured with a Veeco Dektak 8 Stylus Profilometer applying 3 mg force.

### Triboelectric Measurements

The carbon paper was glued on conducting Al tape attached to acrylate holders and contacted against fluorinated ethylene propylene (FEP) film (thickness ∼131 μm) as a tribonegative counter electrode. A setup consisting of a mechanical shaker (S510575, TIRA) connected to a power amplifier (Type BAA120) and function generator (AFG3022, Tektronix) was used to provide mechanical vibrations for the TENG device to undergo contact-separation movements. The output voltage was recorded with an oscilloscope (DPO 3052, Tektronix) connected to a voltage probe (40 MΩ). The short-circuit current (*I*_sc_) was measured with an electrometer (6514, Keithley). The measurements were conducted at 22 °C and 20–23% relative humidity.

## Supporting Information Available

(1) AFM images of MXenes (Figure S1); (2) XRD pattern of MAX and MXenes (Figure S2); (3) Raman and FTIR spectra of MXenes (Figure S3); (4) XPS spectra of MXenes (Figure S4); (5) Chemical composition of Ti_3_CNT_*x*_ materials (Table S1); (6) Chemical composition of Ti_3_C_2_T_*x*_ materials (Table S2); (7) SEM images of Ti_3_C_2_T_*x*_ composites (Figure S5); (8) SEM images of the substrate and Ti_3_CNT_*x*_ composite (Figure S6); (9) SEM image of Ti_3_CNT_*x*_ layer (Figure S7); (10) Digital photographs of MXene composite films (Figure S8); (11) FTIR spectra of Ti_3_CNT_*x*_ materials (Figure S9); (12) Topography and surface potential images (Figure S10); (13) Surface roughness analysis (Figure S11); (14) Triboelectric voltage of plasma treated MXene composites (Figure S12) (PDF)
