# Supplementary material for: Influence of MXene Composition on Triboelectricity of MXene-Alginate Nanocomposites
Source: ACS Appl Mater Interfaces. 2024 Apr 29;16(18):23948–59. doi: 10.1021/acsami.4c03298 (PMC11082886; doi:10.1021/acsami.4c03298)
Supplement: Supplementary file 1 — am4c03298_si_001.pdf [file am4c03298_si_001.pdf]

Supporting Information

Influence of MXene Composition on Triboelectricity of MXene-Alginate Nanocomposites

Bernd Wicklein[1,2,], Geetha Valurouthu[3], HongYeon Yoon[4], Hyunjoon Yoo[2], Sathiyanathan Ponnan[2], Manmatha Mahato[2], Jiseok Kim[2], Syed Sheraz Ali[2], Jeong Young Park[4], Yury Gogotsi[3], Il-Kwon Oh[2,*]*

[1] Materials Science Institute of Madrid (ICMM), Consejo Superior de Investigaciones Científicas (CSIC), 28049 Madrid, Spain.

[2] National Creative Research Initiative for Functionally Antagonistic Nano-Engineering, Department of Mechanical Engineering, Korea Advanced Institute of Science and Technology (KAIST), Daejeon, 34141, Republic of Korea.

[3] Department of Materials Science & Engineering, and A.J. Drexel Nanomaterials Institute, Drexel University, Philadelphia, Pennsylvania, 19104, USA.

[4] Department of Chemistry, Korea Advanced Institute of Science and Technology (KAIST), Daejeon, 34141, Republic of Korea.

* Correspondence and requests for materials should be addressed to I.-K. Oh (Email: ikoh@kaist.ac.kr), B. Wicklein (Email: bernd@icmm.csic.es)

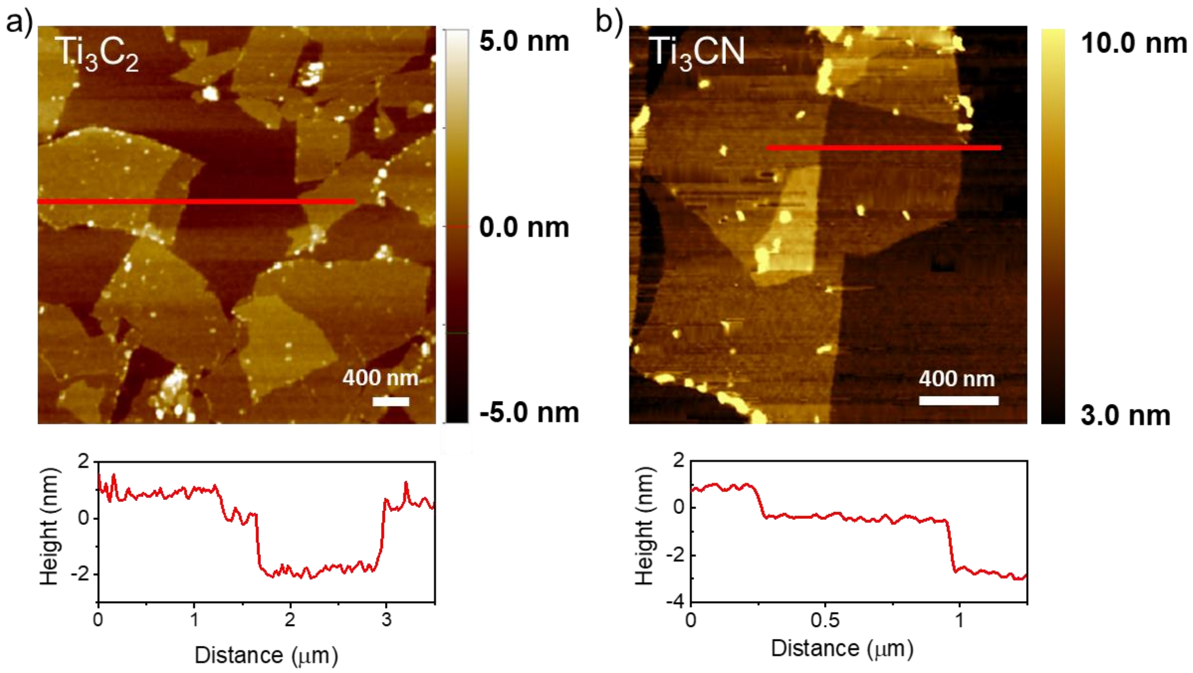

Figure S1. Atomic force microscopy images of (a) $Ti_3C_2T_x$ and (b) Ti_3CNT_x, including height profiles across sheets.

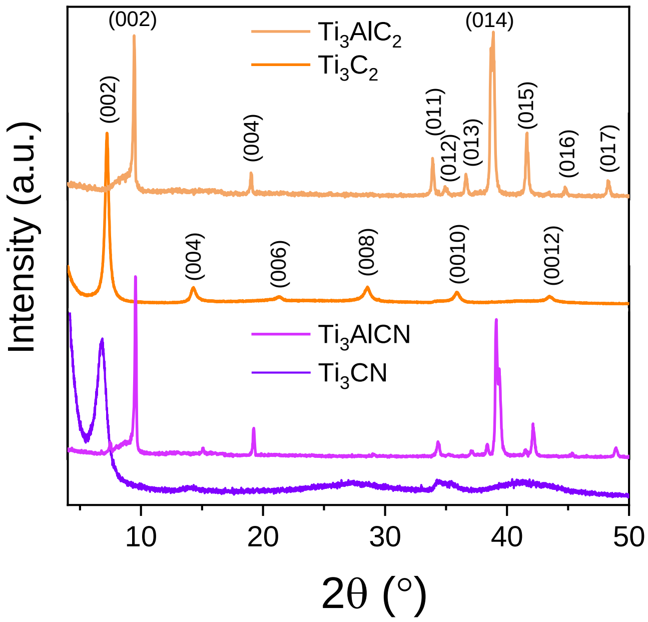

Figure S2. X-ray diffraction pattern of MAX phases and the corresponding MXenes, $Ti_3C_2T_x$ and Ti_3CNT_x.

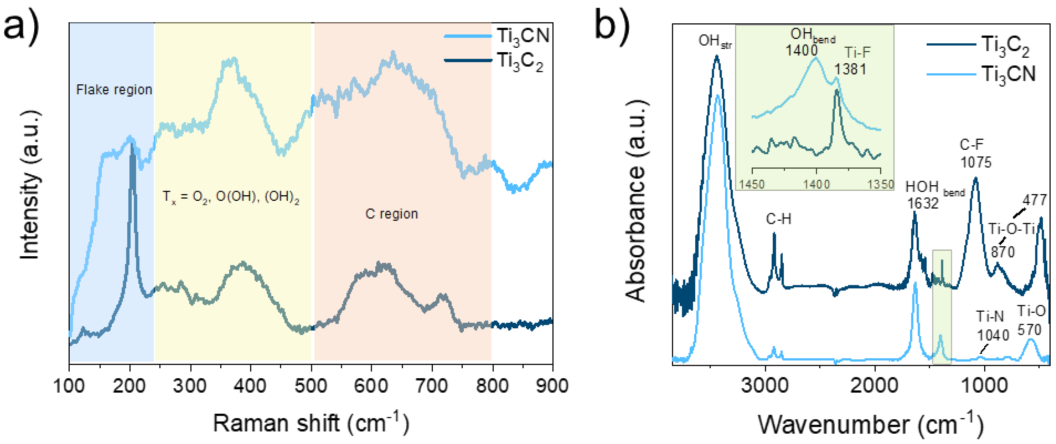

Figure S3. (a) Raman spectra (633 nm excitation wavelength) and (b) FTIR spectra of $Ti_3C_2T_x$ and Ti_3CNT_x.

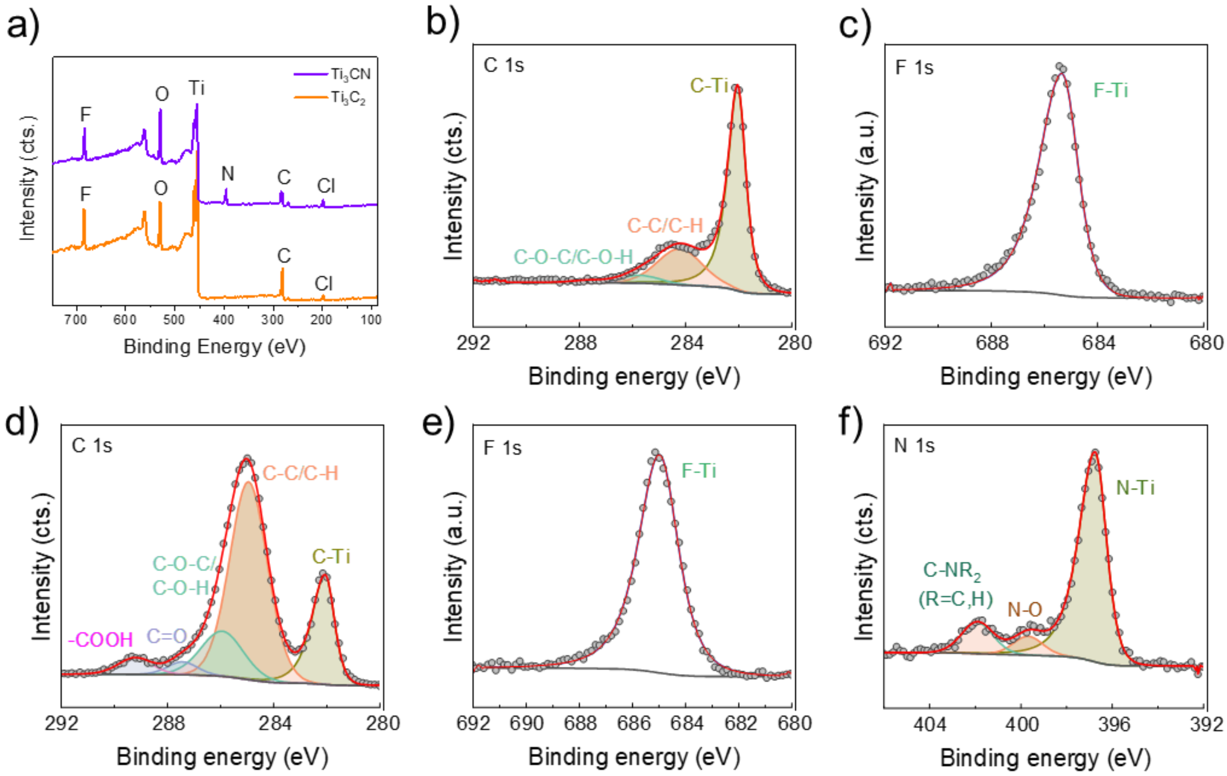

Figure S4. X-ray photoelectron spectra of $Ti_3C_2T_x$ and Ti_3CNT_x. Survey spectra (a), core level C1s (b), F1s (c) spectra of $Ti_3C_2T_x$ and core level C1s (d), F1s (e) and N1s (f) spectra of Ti_3CNT_x.

Table S1. Chemical composition (at%) of Ti_3CNT_x and Ti_3CN-alginate, as determined by XPS analysis.

All			Ti_3CNT_x			Ti_3CNT_x/Alginate		
Region	component	Line shape	Position (eV)	FWHM (eV)	%At conce	Position (eV)	FWHM (eV)	%At conce
Na 1s	COONa	GL(30)	-	-	-	1071.07	1.54	8.74
	Na KLL Auger	GL(30)				535.32	2	3.00
F 1s	F-Ti	LA(1.8,2.2,6)	684.98	1.69	9.03	683.82	1.49	0.37
O 1s	O*-(C=O)-C	GL(30)	-	-	-	532.81	1.7	11.1
	O-(C=O*)-C	GL(30)	-	-	-	530.92	1.4	11.1
	C/Ti-O-H	GL(30)	533.06	2.2	6.14	532.5	1.22	6.12
	C-O-C	GL(30)	-	-	-	531.79	1.37	6.12
	O-Ti $_{oxide}$	GL(30)	530.94	1.37	4.88	529.71	1.41	1.92
	O-Ti $_{fcc}$	GL(30)	532.2	1.3	2.73	-	-	-
	O-Ti $_{bridge}$	GL(30)	529.93	1.2	6.11	-	-	-
Ti 2p	C-Ti-O $_{3/2}$	LA(2,4,6)	455.25	1	3.88	452.17	1.1	0.02
	C-Ti-O $_{1/2}$	LA(2,4,6)	461.25	1.21	1.94	458.17	1.5	0.03
	C-Ti-O,F $_{3/2}$	LA(2,4,6)	456.13	1.2	4.09	454.26	1.2	0.03
	C-Ti-O,F $_{1/2}$	LA(2,4,6)	462.13	1.51	2.05	460.26	1.5	0.04
	C-Ti-F $_{3/2}$	LA(2,4,6)	457.28	1.5	3.9	456.31	1.5	0.07
	C-Ti-F $_{1/2}$	LA(2,4,6)	463.28	1.84	1.95	462.31	2.0	0.03
	$Ti^{4+}{}_{3/2}$	GL(30)	459.37	2	2.88	458.21	1.27	0.39
	$Ti^{4+}{}_{1/2}$	GL(30)	465.17	2.5	1.44	464.01	2.11	0.20
N 1s	N-Ti	LA(2,4,6)	396.75	1.19	5.81	397	1.5	0.04
	N-O	GL(30)	399.69	1.54	0.51	399.59	1.74	0.6
	Amine, C-NR2	GL(30)	401.9	1.63	0.9	402.1	2	0.06
	(R=C,H)							2.84
C 1s	C-Ti	LA(2,4,6)	282.06	0.84	8.74	282.43	1	0.5
	C-C/C-H	GL(30)	284.95	1.67	23.86	284.59	1.38	17.43
	C-OH, C-OC	GL(30)	285.94	1.88	6.11	286.18	1.26	19.51
	C=O	GL(30)	287.44	1.33	1.24	287.73	1.28	9.74
	O-C=O	GL(30)	289.21	1.5	1.8	289.07	1.5	2.84

Table S2. Chemical composition (at%) of $Ti_3C_2T_x$ and Ti_3C_2-alginate, as determined by XPS analysis.

All			$Ti_3C_2T_x$			$Ti_3C_2T_x$/Alginate		
Region	component	Line shape	Position (eV)	FWHM (eV)	%At conce	Position (eV)	FWHM (eV)	%At conce
Na 1s	COONa	GL(30)	-	-	-	1072.05	1.57	8.07
	Na KLL Auger	GL(30)				536.38	2	2.64
F 1s	F-Ti	LA(2,4,6)	685.33	1.44	11.4	684.81	1.31	0.30
O 1s	O*-(C=O)-C	GL(30)	-	-	-	533.78	1.7	11.78
	O-(C=O*)-C	GL(30)	-	-	-	531.9	1.48	11.78
	C/Ti-O-H	GL(30)	533.41	2.5	3.66	533.52	1.11	6.39
	C-O-C	GL(30)	-	-	-	532.9	1.25	6.39
	O-Ti $_{oxide}$	GL(30)	530.89	1.33	8.35	530.75	1.19	0.71
	O-Ti $_{fcc}$	GL(30)	531.83	1.5	5.36	-	-	-
	O-Ti $_{bridge}$	GL(30)	530.09	1.13	5.57	530.12	1.5	0.46
Ti 2p	C-Ti-O $_{3/2}$	LA(2,4,6)	455.16	0.98	8.87	454.94	1.2	0.12
	C-Ti-O $_{1/2}$	LA(2,4,6)	461.16	1.26	4.44	460.94	1.02	0.06
	C-Ti-O,F $_{3/2}$	LA(2,4,6)	456.11	1.22	5.95	455.91	1.4	0.08
	C-Ti-O,F $_{1/2}$	LA(2,4,6)	462.11	1.58	2.98	461.91	1	0.04
	C-Ti-F $_{3/2}$	LA(2,4,6)	457.3	1.5	5.44	457.05	1.48	0.05
	C-Ti-F $_{1/2}$	LA(2,4,6)	463.3	1.87	2.72	463.05	1.09	0.03
	$Ti^{4+}{}_{3/2}$	GL(30)	459.44	2	2.71	459.24	1.53	0.21
	$Ti^{4+}{}_{1/2}$	GL(30)	465.24	2.5	1.36	465.04	2.45	0.11
C 1s	C-Ti	LA(1.4,2,6)	282.04	0.74	20.04	282.1	1	0.44
	C-C/C-H	GL(30)	284.21	2	7.33	285.51	1.3	16.2
	C-OH, C-OC	GL(30)	285.7	1.78	1.42	287.13	1.19	21.94
	C=O	GL(30)	287.7	1.5	0.32	288.69	1.3	10.8
	O-C=O	GL(30)	-	-	-	289.86	1.49	1.4
Cl 2p	Ti-Cl $_{3/2}$	LA(1.8,2,6)	199.44	1.23	1.39	-	-	-
	Ti-Cl $_{1/2}$	LA(1.8,2,6)	201.04	1.13	0.7	-	-	-

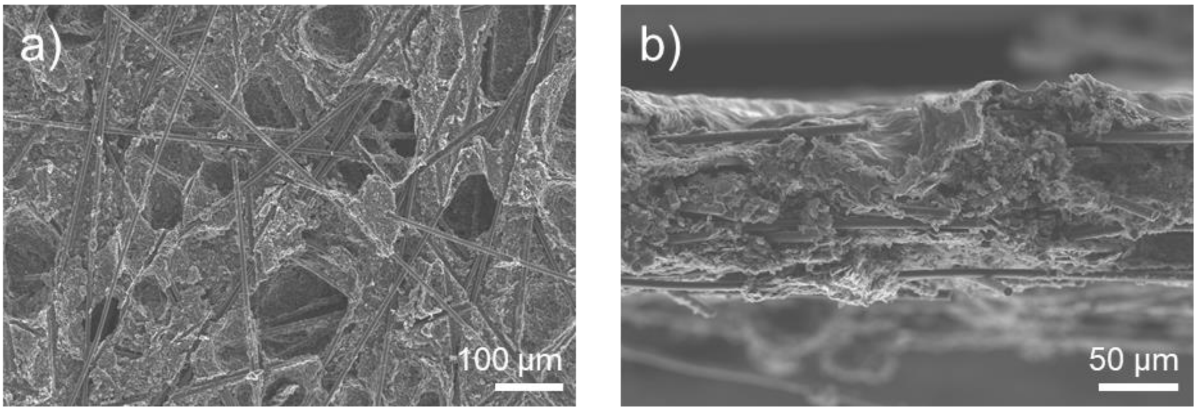

Figure S5. SEM surface (a) and cross-section (b) images of $Ti_3C_2T_x$-alginate coated on carbon fiber paper.

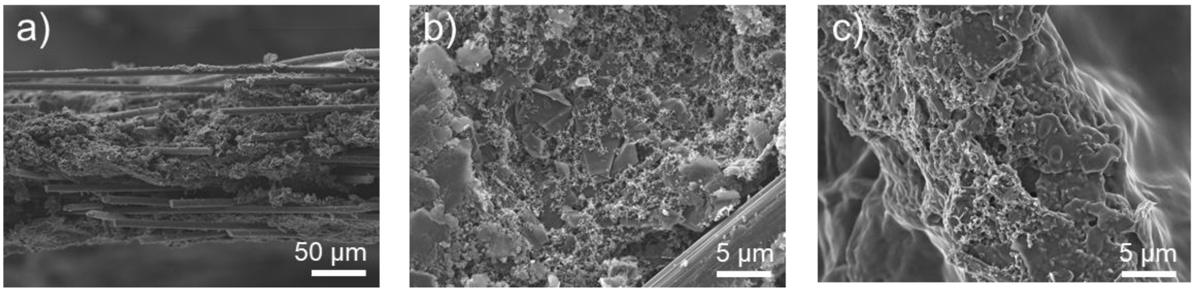

Figure S6. SEM images of the cross-section (a) and surface (b) of O_2 plasma-activated carbon fiber paper and, for comparison, of a fiber coated with Ti_3CN-alginate (c).

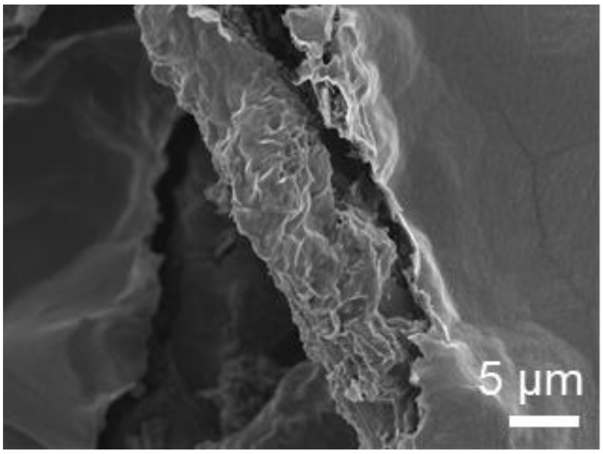

Figure S7. SEM image of a Ti_3CNT_x layer drop cast on carbon fiber paper.

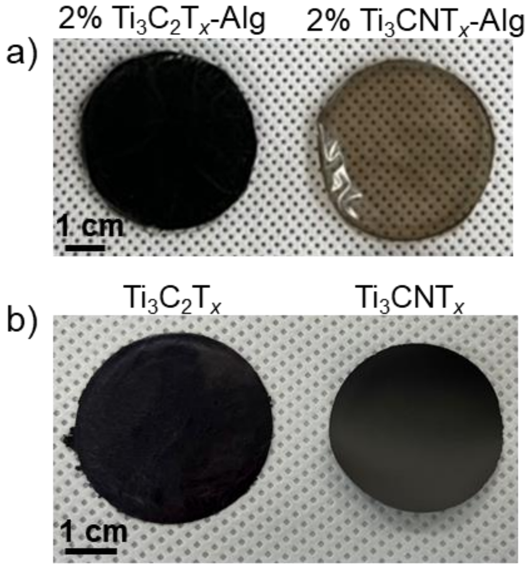

Figure S8. Digital photographs of solvent-cast $Ti_3C_2T_x$-Alg and Ti_3CNT_x-Alg films (a) and vacuum-filtered $Ti_3C_2T_x$ and Ti_3CNT_x films (b).

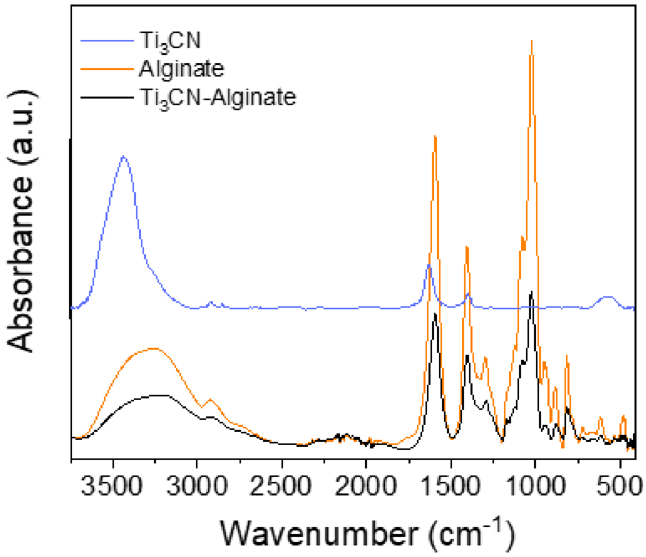

Figure S9. FTIR spectra of Ti_3CNT_x, alginate and Ti_3CN-alginate.

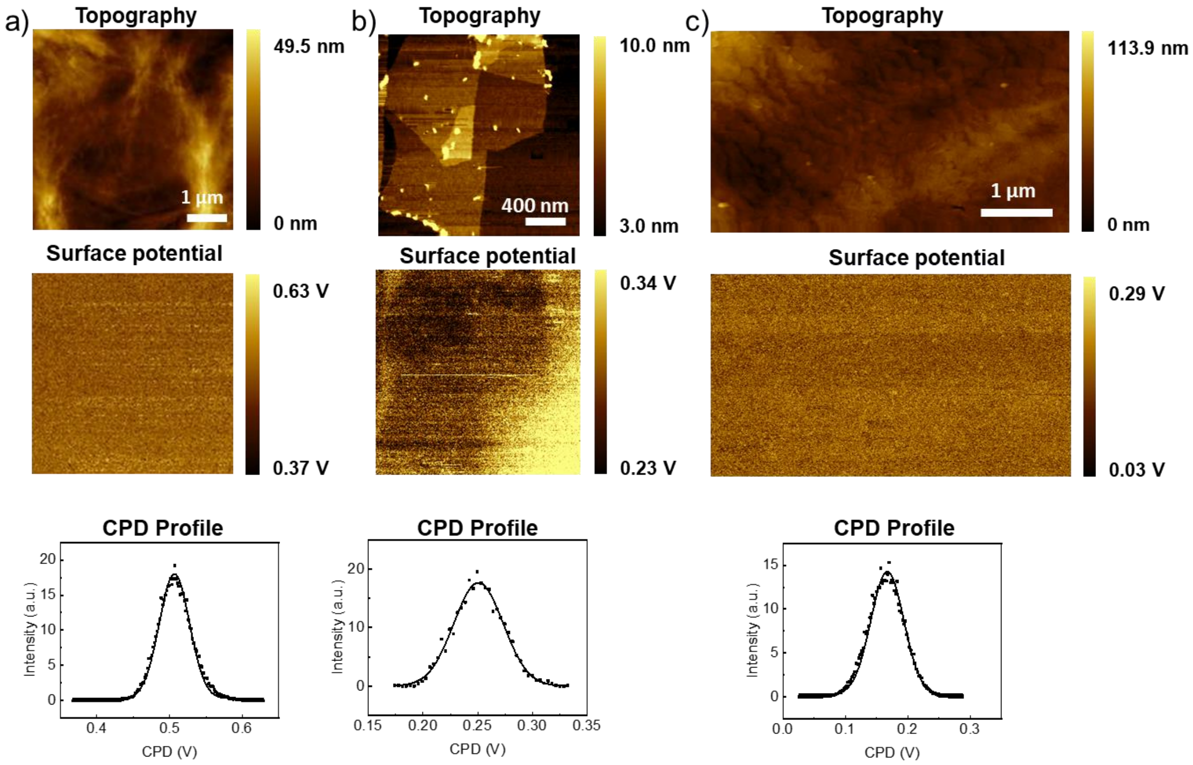

Figure S10. AFM topography, surface potential images and CPD distribution curves of alginate (a), Ti_3CNT_x (b) and Ti_3CNT_x-alginate (c).

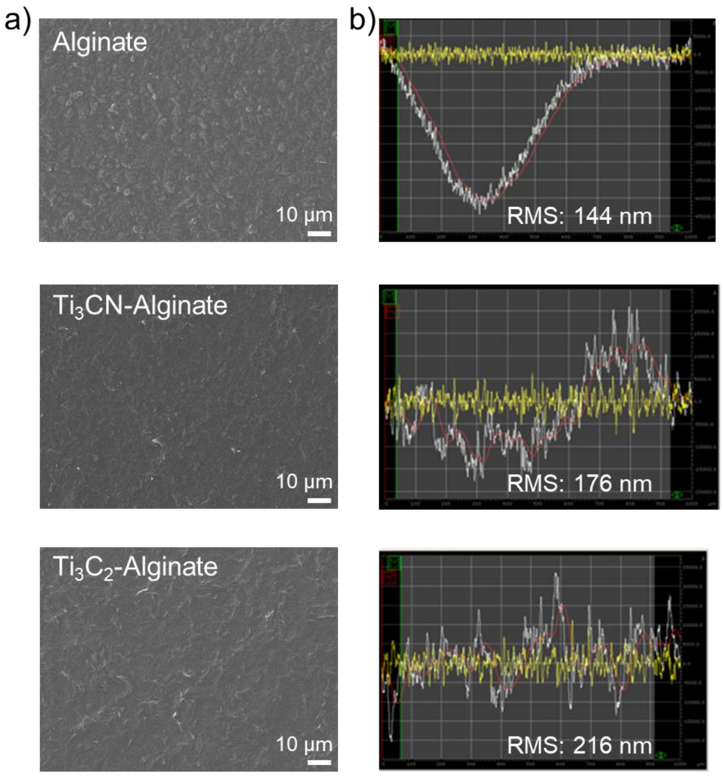

Figure S11. SEM images (a) and 2D profiles (b) of the surface of alginate, Ti_3CNT_x-alginate and $Ti_3C_2T_x$-alginate films. The white curve is the recorded profile and the yellow curve represents the surface roughness after fitting (red line) with a cutoff 25/80 in the software profile "Hills & Valley".

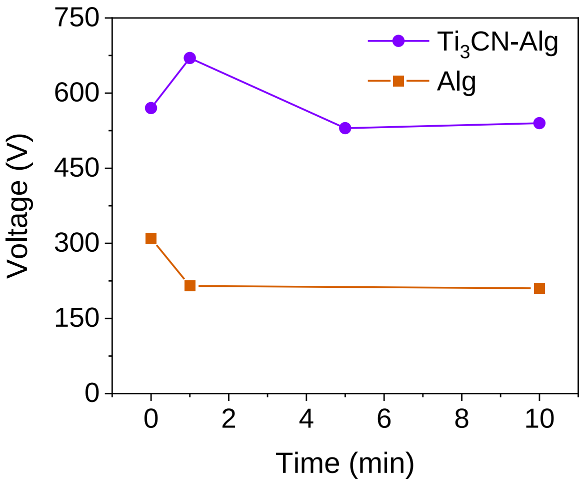

Figure S12. Triboelectric voltage of Ti_3CN-alginate and alginate, respectively, as function of O_2 plasma treatment time.
